# Cannabinoid hyperemesis should be recognised as an effect of chronic cannabis abuse

**Published:** 2014

**Authors:** Sauid Ishaq, Sanaa Ismail, Saad Ghaus, Roop-e-Zahra, Kamran Rostami

**Affiliations:** 1 Gastroenterology Department, Russells Hall Hospital, Dudley, Birmingham City University UK; 2 Gastroenterology Department, Worcestershire Royall Hospital, Worcestershire UK

**Keywords:** Cannabinoid hyperemesi, Endocannabinoids, Thermoregulatory

## Abstract

Here we describe the second reported case of cannabinoid hyperemesis in UK. A 42 years old patient presented on more than one occasion with vomiting, abdominal pain, fever and dehydration and treated as sepsis with antibiotics. Extensive investigations including upper GI endoscopy, colonoscopy, chest X-ray, abdominal ultrasound, abdominal CT scan, barium swallow and echocardiogram; all reported normal. Once the diagnosis of cannabinoid hyperemesis was established, he was advised to abstain from cannabis use resulting in complete resolution of his symptoms.

## Introduction

 A plethora of conditions may cause severe nausea and vomiting in adults. Organic disorders of the gastrointestinal system are often considered first, requiring investigations and imaging techniques. There is a lack of awareness of functional causes of chronic vomiting. Cannabinoid hyperemesis is an adverse effect of chronic cannabis abuse. Patients present with chronic intermittent episodes of severe vomiting, often requiring hospitalisation for treatment of dehydration. There are often associated symptoms of abdominal pain, autonomic dysfunction and curious compulsive hot water bathing behaviour. Indeed, the disorder is often confused with psychiatric illness. It is likely that the syndrome is more common than previously thought, despite the paucity of reports in the medical literature. 

Failure to consider the diagnosis in patients who are chronic cannabis abusers may result in unnecessary gastrointestinal investigations, a delay in diagnosis and continued morbidity. Importantly, all symptoms resolve completely with abstinence from cannabis. We describe the second reported case of cannabinoid hyperemesis in the UK (1).

## Case Reports

A 42 year old gentleman presented with a two day history of profuse vomiting, abdominal pain and accompanying symptoms of feeling feverish, shaking and sweating. He was tachycardic and dehydrated. Systemic examination was unremarkable. Full blood count and inflammatory markers were normal. Urea and creatinine were raised (Urea 17.0 mmol/L (normal range 2.5-7.5), creatinine 120 umol/L (normal range 60-115). Toxicology screen was positive for Cannabis. He had an extensive past medical history of similar episodes of nausea and vomiting, which led to his hospitalisation eight times over the last twenty years. Extensive investigations have been undertaken in the past; upper GI endoscopy, colonoscopy, chest X-ray, abdominal ultrasound, abdominal CT scan, barium swallow and echocardiogram; all reported normal. He is a known cannabis abuser, having been smoking cannabis since 14 years of age, with a total intake of 294 joint years. 

The patient was managed supportively with IV fluids and anti-emetics. Bizarrely, the patient stated that frequent hot showers help to relieve his symptoms. Indeed, his compulsive showering behaviour was commented on by ward staff. It was this odd cluster of symptoms, cyclic nausea and vomiting accompanied by frequent hot water showering presenting in a patient with chronic cannabis abuse that lead to a diagnosis of cannabinoid hyperemesis. He was counselled by drug liaison team and strongly advised to abstain from cannabis use. On this occasion patient was not screened for other narcotic. At an outpatient appointment 3 months post-discharge, he had managed to abstain from cannabis use and was completely symptom-free.

## Discussion

In 1882 Samuel Gee first reported the occurrence of cyclic vomiting syndrome (CVS) in children (2). The validity of this syndrome has been questioned (3, 4).

A characteristic triad is suggestive of cannabinoid hyperemesis; chronic cannabis abuse, cyclic vomiting and compulsive bathing behaviour, although more stringent characteristics for the diagnosis of the syndrome have been recently proposed (5). A stereotypical clinical course has been identified, commencing with a prodromal phase during which patients suffer from nausea particularly in the morning. The severity of symptoms increases thereafter, with patients suffering from recurring episodes of nausea, continuous vomiting and colicky abdominal pain (6). Patients have also reported, as in our case, feeling sweaty or feverish. This can be perceived as pointing to an infective or inflammatory cause. On previous admissions our patient was given intravenous antibiotics, quite unnecessarily. Reports in the literature also demonstrate that patients with cannabinoid hyperemesis have previously been diagnosed as having psychogenic vomiting (7).

Cannabis is a common drug of abuse that is associated with several pathological and psychiatric adverse effects. It has also, however, found use for medicinal purposes. Traditionally, cannabis has been associated with an antiemetic action, and has been used therapeutically to treat chemotherapy-induced nausea and vomiting, as well as in a range of other clinical contexts (8). This anti-emetic effect of endocannabinoids is largely mediated by type 1 cannabinoid (CB1) receptors in the brain and intestinal nerve plexus, although some of their effect may also be receptor dependent (5).

This syndrome thus presents the paradoxical effect of hyperemesis in chronic cannabis users. Such a paradoxical response has only previously been demonstrated with acute toxicity to intravenous injection of crude marijuana extract (8). The pathophysiology underlying cannabinoid hyperemesis is unknown, and proposed mechanisms include toxicity due to marijuana’s long half-life, fat solubility, delayed gastric emptying, and thermoregulatory and autonomic disequilibrium via the limbic system (5). Cannabinoids are known to impair peristalsis in a dose-dependent manner (5), and current research has identified type 1 cannabinoid receptors in the intestinal tract that have an inhibitory effect on gastrointestinal motility (9). These peripheral effects of cannabinoids can theoretically override the centrally mediated anti-emetic effects, resulting in hyperemesis (5).

It is important to note that the compulsion to take frequent hot showers or baths displayed in patients with cannabinoid hyperemesis is not psychiatric in origin. It is a learned behaviour, which has been found to be absent in patients with the first few episodes of illness, but once established rapidly becomes a compulsion (7). Symptomatic relief, with the alleviation of nausea, vomiting and abdominal pain, is temperature dependent, with the greatest effect with hotter water. Indeed, there are reports in the literature of patients scalding themselves in an attempt to get the water as hot as possible (7). It has been proposed that the thermoregulatory role of endocannabinoids may be responsible for this peculiar feature (9). Two proposed mechanisms are that the brain may react to changes in core body temperature due to the dose-dependent hypothermic effects of delta-9-tetrahydrocannabinol (the psychoactive component of cannabinoid) (10). Alternatively, the bathing behaviour may be a result of direct CB1 receptor activation in the hypothalamus by delta-9-tetrahydrocannabinol or another active compound and may not necessarily be a response to changes in core body temperature (9).

It remains clear that chronic cannabis abuse is the key factor in the development of cannabinoid hyperemesis. Both this case and reports in the literature show that patients improve in the months following discontinuation of cannabis use, confirming the diagnosis. Appropriate patient follow-up is thus essential. During the acute phase, treatment consists only of supportive measures (11), cyclic vomiting of cannabinoid hyperemesis is reported to be resistant to a wide range of antiemetics (6). Confirmation of cannabis abuse as causative in a patient’s illness may provide them with motivation to abstain from the illicit drug, as was the case in this patient. Prompt recognition of cannabinoid hyperemesis is thus rewarded with a clear path for management, which is often a life-changing event for affected patients.

Chronic cannabis abuse, therefore, is a cause of a debilitating syndrome resembling cyclic vomiting syndrome. There is very little published data available on cannabinoid hyperemesis. This in our view is due to the lack of awareness and recognition of the condition, and its confusion with disorders of a psychiatric aetiology. Clinicians should be aware of cannabinoid hyperemesis as a diagnosis for cyclic vomiting in a setting of chronic cannabis abuse. This would result in fewer hospital admissions and needless investigations, and may provide patients with real motivation to abstain from cannabis. Given the prevalence of illicit cannabis use both in the UK and internationally, and the continuing use of cannabis for therapeutic purposes, this condition and other effects of cannabinoids require further investigation.
